# Cytoreductive Surgery and Perioperative Intraperitoneal Chemotherapy Experience in Peritoneal Carcinomatosis: Single-Center Analysis of 180 Cases

**DOI:** 10.1155/2021/8851751

**Published:** 2021-04-22

**Authors:** Kursat Karadayi, Meric Emre Bostanci, Murat Can Mollaoglu, Ufuk Karabacak

**Affiliations:** Department of Surgical Oncology, Cumhuriyet University Faculty of Medicine, Sivas, Turkey

## Abstract

**Background:**

In peritoneal carcinomatosis (PC), increased life span and disease-free survival times are shown in patients with hyperthermic intraperitoneal chemotherapy (HIPEC) and early postoperative intraperitoneal chemotherapy (EPIC) following cytoreductive surgery (SRC). In this study, our main objective was to present our experience of performing SRC and perioperative intraperitoneal chemotherapy (HIPEC and EPIC) on patients with PC, in light of the literature.

**Methods:**

Demographic data, follow-up results, peritoneal carcinomatosis index (PCI), completeness of cytoreduction (CCR) score, and morbidity and mortality rates of 180 patients treated with SRC + HIPEC + EPIC for PC at the Department of Surgical Oncology at Sivas Cumhuriyet University between January 2008 and July 2020 were analyzed retrospectively.

**Results:**

Distribution of 180 PC cases according to primary organs included 53 ovarian, 39 colorectal, 33 stomach, 25 primary peritoneum, 10 uterus, 10 tuba, five soft tissue, and five appendix originated carcinoma. The average PCI of the cases detected preoperatively was 21 (5–30). Completeness of cytoreduction scores of CCR-0 in 102 cases, CCR-1 in 67 cases, CCR-2 in eight cases, and CCR-3 in three cases was obtained. Median operation time was 300 (200–540) minutes. Perioperative morbidity rate was 47.0%, and perioperative mortality rate was 13.5%.

**Conclusion:**

The peritonectomy procedure is a difficult, long-lasting, troublesome intervention, but it is the most important treatment option with acceptable morbidity and mortality rates in patients selected for PC treatment in experienced centers.

## 1. Background

Peritoneal carcinomatosis (PC) may present as primary tumors of the peritoneum or as metastases of gynecological and gastrointestinal tumors to the peritoneum. Life expectancy of those with PC is short (average 3–6 months); however, better survival results were obtained with the peritonectomy procedure [hyperthermic intraperitoneal chemotherapy (HIPEC) and early postoperative intraperitoneal chemotherapy (EPIC) following cytoreductive surgery (SRC)] as defined by Sugarbaker [1, 2]. Even though the morbidity and mortality rates from the procedure are still high, it has become feasible in many centers due to an increase in experience among surgeons and increased technological developments [3]. Our aim in this study is to discuss and share the data we obtained when applying the peritonectomy procedure in PC treatment, in light of the literature.

## 2. Methods

Between January 2008 and July 2020, the data of 180 patients who underwent the peritonectomy procedure due to PC at the Department of Surgical Oncology, Sivas Cumhuriyet University, were retrospectively recorded and analyzed. Routine blood tests and tumor markers were studied in all patients. Thoracoabdominal computed tomography, laparoscopy, and positron emission tomography (PET-CT) after March 2012 were used for staging. Cases were evaluated in terms of age, gender, primary tumor origin, duration of surgery, PCI, CCR score, resection types, perioperative complications (Clavien Dindo classification), and mortality. Patients with extra-abdominal metastasis, a history of abdominal radiotherapy, massive small intestine and mesentery involvement, massive gastrohepatic ligament involvement, and poor performance were excluded from the study.

### 2.1. Surgical Technique

**Figure 1:**
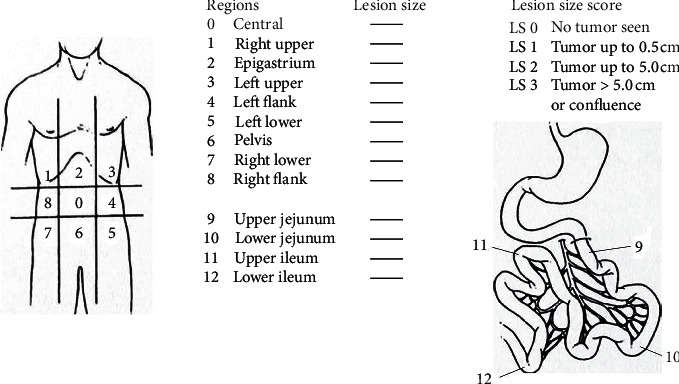
Peritoneal cancer index [4].

**Figure 2:**
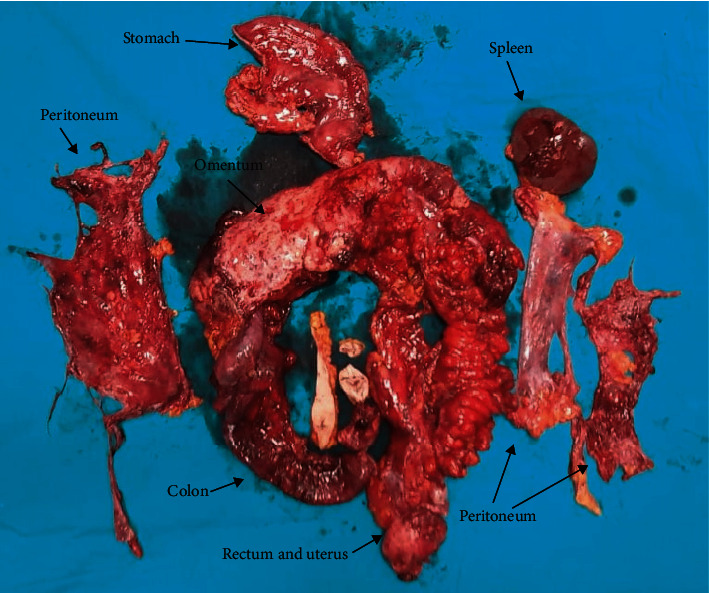
Surgically resected tissues.

The operations were performed with a midline incision made from xiphoid to pubis. Ascites or gelatinous fluid decompression was performed. The PCI, as described by Sugarbaker, was calculated and recorded (Figure 1) [4]. In addition to peritonectomy, all tumoral tissues and organs were resected for complete cytoreduction (Figure 2). In tumors of gynecological origin, paraaortic and pelvic lymph node dissection was additionally performed. At the end of the surgery, CCR score was calculated and recorded. According to this classification system, CCR-0 means a macroscopic tumor that is completely resected, CCR-1 means a residual tumoral lesion less than 2.5 mm, CCR-2 means a residual tumoral lesion in the range of 2.5 mm to 2.5 cm, and CCR-3 means residual tumors larger than 2.5 cm, indicating the presence of a tumoral lesion. CCR-0 and CCR-1 were considered as complete cytoreduction.

### 2.2. HIPEC and EPIC Procedure

The abdomen was closed after placing two inflow and two outflow lines and heat probes in the abdomen. HIPEC was performed using the closed method. For 60 minutes, chemotherapeutic agents were administered intraperitoneally in a 3000 cc 0.9% saline solution at a temperature of 41°C–43°C. EPIC was administered intraperitoneally normothermic between the first day and the fifth day postoperatively. Chemotherapeutic agents delivered from inflow lines were kept in the abdomen for 23 hours. At the end of the 23 hours, the outflow lines were opened, and the perfusate fluid was removed. The administration of EPIC was completed in five days by applying the same procedure for four consecutive days (Table 1).

### 2.3. Statistical Analysis

The data obtained from the raw data were recorded in the IBM SPSS Statistics 22 (IBM SPSS, Turkey) program, and the distribution of the data in the average mode, median, and percentile slices were calculated and recorded in tables, which are portrayed in the results section. The Kaplan–Meier Curve and the Logrank tests were used for survival analysis.

## 3. Results

**Figure 3:**
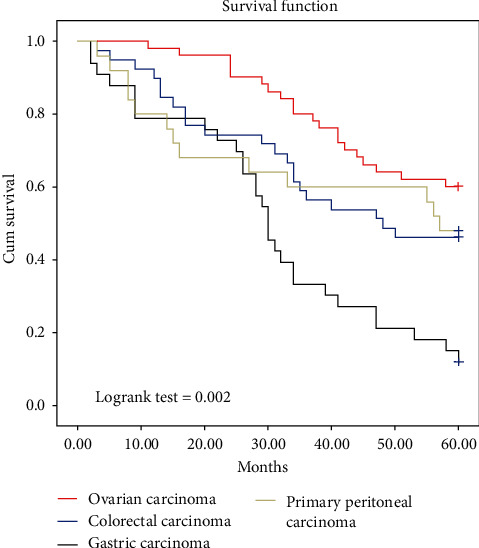
Survivability of study population.

The mean age of the patients was 62 (±14.7) years; 119 (66.1%) of the cases were female, and 61 (33.8%) were male. Distribution of 180 PC cases according to primary organs included 53 ovarian, 39 colorectal, 33 stomach, 25 primary peritoneum, 10 uterus, 10 tuba, five soft tissue, and five appendix originated carcinoma. The average PCI of the cases detected preoperatively was 21 (5–30). CCR score of CCR-0 in 102 cases, CCR-1 in 67 cases, CCR-2 in eight cases, and CCR-3 in three cases were obtained. Median operation time was 300 (200–540) minutes (Table 2). Organ and tissue resections are shown in Table 3. A total of 156 gastrointestinal and colonic anastomoses were performed in 90 patients. The number of patients with temporary stoma was 62, and the number of patients with permanent stoma was ten. HIPEC was applied to all patients; EPIC was applied to 140 patients. The mean intraoperative erythrocyte suspension and fresh frozen plasma transfusion were 2.0 (1–6) and 1.5 (1–3) units, respectively. The average length of hospital stay was 12 (8–38) days, and the average length of stay at the intensive care unit was three (1–18) days. Perioperative morbidity was seen in 80 (47.0%) patients. The main causes were superficial surgical site infection (23), paralytic ileus (13), gastric atony (14), anastomosis leak (17), pulmonary embolism (8), intraabdominal abscess (5), and pleural effusion (18). Hematological toxicity (18), renal toxicity (15), intraabdominal fluid collection (6), lymphatic leak (7), bile leak (4), and evisceration/eventration (8) were seen in the patients (Table 4). Mortality was observed in 23 patients (13.5%) in the early period. Of these 23 patients, six died due to cardiac failure, four died due to pulmonary failure, three died due to renal failure, six died due to anastomosis leaks, and four died due to sepsis. Intraabdominal recurrence occurred in 13 patients, five of them originating from ovarian, four originating from peritoneum, and four originating from colon-rectum; in six patients, recurrence occurred in flow locations. Secondary cytoreduction was performed in all 19 patients. Liver metastasis was detected in two patients with ovarian cancer and two patients with colorectal cancer in the first 12 months, postoperatively; metastasectomy was subsequently performed in these patients. All 143 patients without perioperative mortality were followed up with adjuvant chemotherapy. The follow-up and treatment of 56 cases continued, and our case, which has the longest follow-up period, is at its 84th month. Our five-year survival data (Kaplan–Meier survival analysis) is given in Figure 3. In the Logrank test, the estimated overall survival was found to be 48.8 (95% CI, 46.72–50.98 ) months.

## 4. Discussion

The peritonectomy procedure is complex, involving intraperitoneal chemotherapy (HIPEC + EPIC) together with SRC in PC treatment. SRC is a troublesome procedure for a long-term surgeon, especially in low-performance patients who have undergone multiple abdominal surgeries and received chemotherapy before surgery [5]. The main goal of SRC is to provide complete cytoreduction (CCR-0/1). For this, additional multivisceral resections may be required for peritonectomy [6]. Peritonectomy should be carried out in full for primary peritoneal tumors. It is suggested that, outside of the primary peritoneal tumors, the removal of the tumor-capped peritoneum is sufficient in PC patients [2, 7, 8]. In our series, total peritonectomy was performed in all 25 primary peritoneal patients. In patients with PC other than primary peritoneum, peritonectomy was performed according to the prevalence of the peritoneum.

The most important treatment option in the treatment of malignant peritoneal mesothelioma (MPM) was SRC + HIPEC + EPIC. Out of 25 patients with primary peritoneal tumors, 20 were MPM. The high number of these cases can be explained by the geographical proximity of our center to *s* where the exposure to asbestos accounts for the most common cause of MPM in the World [5].

One of the tissues most frequently affected by PC is the omentum. The approach of our clinic is to perform a total omentectomy, regardless of how much of the omentum is retained. Omentectomy was completed by connecting and cutting the stomach along the entire great curvature from the entrance of the gastroepiploic vessels to the stomach. The cause of delayed gastric emptying in our series may be the skeletonization of the large curvature of the stomach [3].

In patients with PC, the spleen may be affected as a result of capsule involvement or parenchymal involvement. In this case, splenectomy should be added to the SRC. The positive effects of the spleen on the immunological system are known; therefore, if possible, partial splenectomy should be the preferred procedure [9, 10]. Splenectomy was performed in 41 patients, completely in 36 and partially in five.

In six of our cases, intraabdominal sterile fluid collection developed, and these collections were drained with a percutaneous catheter. Cytological and microbiological evaluations of these cases were reported as negative. This can be explained by the fact that intraperitoneal fluid, which was given due to postoperative early intraabdominal adhesion development, was not sufficiently drained [3, 5].

The colon is one of the affected organs in patients with PC. Colon resection is required when performing SRC due to both massive omental disease and involvement of the colon wall. In our series, 57 patients underwent colorectal resection. This attempt was made in accordance with the principles of total mesorectal excision (TME) and complete mesocolic excision (CME), independent of the primary tumor site. Of these, 39 were primary colorectal tumors, and 28 were noncolorectal tumors. In our series, tumors were detected in the lymph nodes of the colon through histopathological examination in 16 of the PC patients, except in the primary colon. This situation is remarkable, and no relevant information has been found in the literature; therefore, while performing colon resection due to PC, we recommend performing colon resection in accordance with the TME and CME principles, regardless of the origin of the primary tumor.

Whether cytoreduction should be performed in the presence of liver metastasis is still a matter of debate [11–14]. If the application of our clinic is less than four in liver metastasis, limited in one lobe, and less than 2 cm, R0 resection can be achieved by surgical intervention (with metastasectomy, segmentectomy, and radiofrequency ablation (RF) application), and cytoreductive surgery is not considered a contraindication. In our clinic, liver metastasis was detected in 22 patients during the first SRC, and SRC was subsequently performed in these 22 patients (metastasectomy in eight patients, segmentectomy in four patients, and RF ablation in 10 patients). Resection is recommended for liver metastases, especially in recurrent SRCs in ovarian tumors [13]. Four patients underwent metastasectomy during secondary SRC due to metastasis developed after SRC. In two of our patients who underwent metastasectomy, an intraabdominal abscess developed, and percutaneous drainage was performed. The developing abscess may be due to the RF technology used during resections and the associated small biliary tract leaks [15].

Total abdominal hysterectomy with bilateral salpingo-oophorectomy (TAH + BSO) SRC is one of the resections performed in PC. TAH + BSO was performed in 68 patients in our series. Primary gynecological origin tumors were observed in 78 patients. Of these 78, 45 were primary surgical interventions prior to SRC, followed by PC developed patients. In 33 patients, the PC was simultaneous with the primary tumor. In 23 patients, TAH + BSO was performed due to ovarian, tuba, or uterine metastases of different tumors. In particular, patients of childbearing age who plan to have a baby should be informed about this before surgery.

HIPEC applied during the surgery has many advantages. These include the cytotoxic effect of hyperthermia, facilitating the entry of the chemotherapeutic agent into the tumor cell by increasing permeability in tumor cells, the systemic side effect is less than that of systemic chemotherapy, and the high dose chemotherapeutic agent can be administered to the intraperitoneal cavity [8, 16]. HIPEC can be applied in two ways: the open or closed method. Our clinic applies the closed technique. Among the advantages of the closed technique, we have applied the lower exposure of the operating room staff to the chemotherapeutic agent, the surgeon's need not to be present at the operating room, the increased intraabdominal pressure increasing the tissue penetration of the chemotherapeutic agent, and less intraperitoneal heat loss [8, 16]. Six patients had recurrence at the flow site; this situation may be attributed to the inadequate contact of the flow sites with chemoperfusate during closed HIPEC; this is a disadvantage of closed HIPEC.

Perioperative intraperitoneal chemotherapy (HIPEC ± EPIC) is part of the peritonectomy procedure. However, the HIPEC and EPIC protocol has not been fully standardized in the literature; different applications are performed in different centers [7, 16–21]. In our clinic, following SRC, both HIPEC and EPIC are applied together. In our study, HIPEC was applied to all 180 patients. EPIC, however, could only be applied to 140 patients; it could not be applied to 24 patients, and the fifth day of treatment could not be completed in 16 patients. The reasons for this are postoperative general state indifference and hematological and renal toxicity development. The developing hematological toxicity (neutropenia, thrombocytopenia) can be attributed to both surgical factors and the systemic cytotoxic absorption of HIPEC and EPIC that was administered. Although many factors are to blame, myelosuppression is the most important [22, 23].

The primary factors determining survival rates in those with PC are primary tumor histopathology, PCI, and the CCR score. In our study, the mean PCI was 21, which is a relatively high rate compared to the literature. This situation can be explained by our patient's late administration to our clinic. The main purpose of cytoreductive surgery is to provide complete cytoreduction. CCR should be zero or one in order to achieve complete cytoreduction. To achieve this, multivisceral resections may be required, in addition to peritonectomy. Multivisceral resections are naturally associated with prolonged surgery, excessive blood loss, and increased mortality and morbidity [24].

Although our PCI rate is high, our complete cytoreduction rate is also high. The reason for our success can be explained by the high rate of our extreme cytoreduction. The concept of extreme cytoreduction is defined as five or more major organs (small intestine, colon, rectum, spleen, pancreas, gallbladder, stomach, and full-thickness diaphragm) resection, or three or more intestinal anastomosis [25]. Our mortality rate is 13.5%. The partial elevation of our mortality rate in our series can be attributed to the high number of extreme resections.

After the procedures of cytoreductive surgery and intraperitoneal chemotherapy, the major morbidity rate varies between 20.8% and 53.3% according to various sources [26–28]. This severe morbidity rate, which was 29.4% in our study, is similar to other studies. In our study, the total morbidity rate was calculated as 47.0%. Our higher morbidity rate can be partially attributed to the high PCI and our effort to provide complete cytoreduction. Complete cytoreduction was accomplished in 159 patients. Complete cytoreduction, however, could not be achieved in 21 patients. The reasons for this are a higher PCI than predicted in seven patients before surgery, intraoperative hypotension developing in eight patients, massive gastrohepatic ligament involvement in four patients, and massive small bowel meso-involvement in two patients.

The most common complications observed after cytoreductive surgery are intraabdominal abscess, enterocutaneous fistula, prolonged ileus, pneumonia, and hematological problems [29, 30]. The most common complications in our study were superficial surgical site infection and paralytic ileus. In our series, gastrointestinal anastomosis leaks and gastrointestinal fistula rates were lower than those reported in the literature, which can be explained by our high rate of protective stoma opening [3].

Adhesion after SRC is another problem. This may be due to both extensive dissection, which was previously performed during SRC, and the intraperitoneal chemotherapy given [31]. Secondary cytoreduction is recommended in selected cases [32]; however, this situation should be taken into consideration in patients undergoing secondary SRC, and meticulous surgery should be performed to prevent intraabdominal organ and vascular injury.

In our series, all tumors of appendix origin were pseudomyxoma peritonei (PMP). Cytoreductive surgery + HIPEC + EPIC was applied to all of them. All of the patients are still being followed up without recurrence at 65, 76, 78, 80, and 82 months, respectively. Cytoreductive surgery + EPIC + HIPEC was applied to five of our patients with soft tissue tumors. All of these patients are still being followed up without recurrence at 56, 68, 74, 76, and 82 months, respectively. Our five-year survival rates are 12%, 46%, 48%, and 62% for stomach, colon, peritoneum, and ovarian tumors, respectively.

## 5. Conclusion

Peritonectomy procedures can be performed safely in selected patients and in centers experienced in PC treatment. In order to obtain good survival rates, providing complete cytoreduction should be the main goal, even in patients with high PCI.

## Figures and Tables

**Table 1:** HIPEC/EPIC protocols.

Malignancies	HIPEC	EPIC
Gynecological and primary peritoneum	Mitomycin C 20 mg/m^2^, cisplatin 30 mg/m^2^	Paclitaxel 20 mg/m^2^
Colorectal, gastric, appendix	Mitomycin C 20 mg/m^2^, cisplatin 30 mg/m^2^	5-fluorouracil 650 mg/m^2^
Sarcoma	Mitomycin C 20 mg/m^2^, doxorubicin 15 mg/m^2^	Melphalan 10 mg/m^2^

HIPEC: hyperthermic Intraperitoneal Chemotherapy, EPIC: early Postoperative Intraperitoneal Chemotherapy.

**Table 2:** Patients characteristics.

Age	62 (24–84)
Gender (n)	
Female	119
Male	61
ASA (n)	
1	52
2	72
3	46
PCI score	21 (5–30)
0-10	46
11-20	80
21-30	54
CCR score (n)	
0	102
1	57
2	8
3	3
Length of hospital stay (Day)	12 (8–38)
Length of ICU stay (Day)	3 (1–18)
Clavien–Dindo complication score	
1	31
2	52
3A	47
3B	8
4	9
5	23

ASA: American Society of Anesthesiologists, PCI: peritoneal carcinomatosis index, CCR: completeness of cytoreduction score, ICU: intensive care unit.

**Table 3:** Surgical procedures.

Surgical procedure	(*n*)
Total abdominal hysterectomy + bilateral salpingo-oophorectomy	68
Colorectal	57
Proctocolectomy	10
Total colectomy	20
Subtotal colectomy	10
Right hemicolectomy	8
Left hemicolectomy	4
Anterior resection	5
Stomach	21
Total gastrectomy	10
Subtotal gastrectomy	11
Ileum-jejunum resection	57
Liver	26
Metastasectomy	12
Segmentectomy	4
RF ablation	10
Spleen	41
Total splenectomy	36
Partial splenectomy	5
Cholecystectomy	48
Omentectomy	122
Diaphragm resection	6
Peritoneum	157
Total peritonectomy	94
Pelvic peritonectomy	37
Hemidiaphragmatic striping	20
Anterior parietal peritonectomy	6
Appendix	49
Paraaortic lymph node dissection	78
Adrenalectomy	8
Extreme cytoreduction	116

**Table 4:** Postoperative complications.

Complications	*n*	Treatment
Superficial surgical site infection	23	Medical/local wound care
Paralytic ileus	13	Medical
Gastric atony	14	Medical/nasogastric tube drainage
Anastomosis leak	17	Surgery
Pulmonary embolism	8	Medical
İntraabdominal abscess	5	Percutaneous drainage
Pleural effusion	18	Drainage
Hematological toxicity	18	Medical
Renal toxicity	15	Medical
İntraabdominal fluid collection	6	Percutaneous drainage
Lymphatic leak	7	Medical
Bile leak	4	Medical
Evisceration/Eventration	8	Surgical

## Data Availability

Patient data are based on medical records maintained by our hospital. More detailed information from the data specified in the tables can only be shared with the permission of the Ministry of Health if requested by the editor.
